# Structural Insights into New Bi(III) Coordination Polymers with Pyridine-2,3-Dicarboxylic Acid: Photoluminescence Properties and Anti-*Helicobacter pylori* Activity

**DOI:** 10.3390/ijms21228696

**Published:** 2020-11-18

**Authors:** Mateusz Kowalik, Joanna Masternak, Iwona Łakomska, Katarzyna Kazimierczuk, Anna Zawilak-Pawlik, Piotr Szczepanowski, Oleksiy V. Khavryuchenko, Barbara Barszcz

**Affiliations:** 1 Institute of Chemistry, Jan Kochanowski University in Kielce, Uniwersytecka 7, 25-406 Kielce, Poland; joanna.masternak@ujk.edu.pl (J.M.); barbara.barszcz@ujk.edu.pl (B.B.); 2 Faculty of Chemistry, Nicolaus Copernicus University in Toruń, Gagarina 7, 87-100 Toruń, Poland; 3 Department of Inorganic Chemistry, Faculty of Chemistry, Gdańsk University of Technology, G. Narutowicza 11/12, 80-233 Gdańsk, Poland; katarzyna.kazimierczuk@pg.gda.pl; 4 Laboratory of Molecular Biology of Microorganisms, Microbiology Department, Hirszfeld Institute of Immunology and Experimental Therapy, Polish Academy of Sciences, Weigla 12, 53-114 Wrocław, Poland; anna.pawlik@hirszfeld.pl (A.Z.-P.); szczepanowski.piotr@gmail.com (P.S.); 5 Shupyk National Medical Academy of Postgraduate Education (NMAPE), Dorogozhytska 9, 04112 Kyiv, Ukraine; alexkhavr@gmail.com

**Keywords:** Bi(III) coordination polymers, pyridine-2,3-dicarboxylic acid, holodirected and hemidirected geometry, photoluminescence, *Helicobacter pylori*

## Abstract

Two novel coordination polymers, [Bi_2_(2,3pydc)_2_(2,3pydcH)_2_(H_2_O)]_n_ (**1**) and {(Et_3_NH)_2_[Bi(2,3pydc)(2,3pydcH)Cl_2_]}_n_ (**2**) were prepared using as a prolinker pyridine-2,3-dicarboxylic acid (2,3pydcH_2_). The obtained complexes were fully characterized by elemental analysis, TG/DTG, FT-IR, solid-state photoluminescence, DFT calculations and single-crystal X-ray diffraction. The obtained complexes crystallized in the triclinic *P*-1 space group (**1**) and comprise dimeric units with two crystallographically different Bi(III) centers (polyhedra: distorted pentagonal bipyramid and bicapped trigonal prism) and monoclinic *P*2_1_/*c* space group (**2**) with a distorted monocapped pentagonal bipyramid of Bi(III) center. The various coordination modes of bridging carboxylate ligands are responsible for the formation of 1D chains with **4,5C10** (**1**) and **2C1** (**2**) topology. The photoluminescence quantum yield for polymer **2** is 8.36%, which makes it a good candidate for more specific studies towards Bi-based fluorescent materials. Moreover, it was detected that polymer **1** is more than twice as active against *H. pylori* as polymer **2**. It can be concluded that there is an existing relationship between the structure and the antibacterial activity because the presence of chloride and triethylammonium ions in the structure of complex **2** reduces the antibacterial activity.

## 1. Introduction

**Scheme 1:**
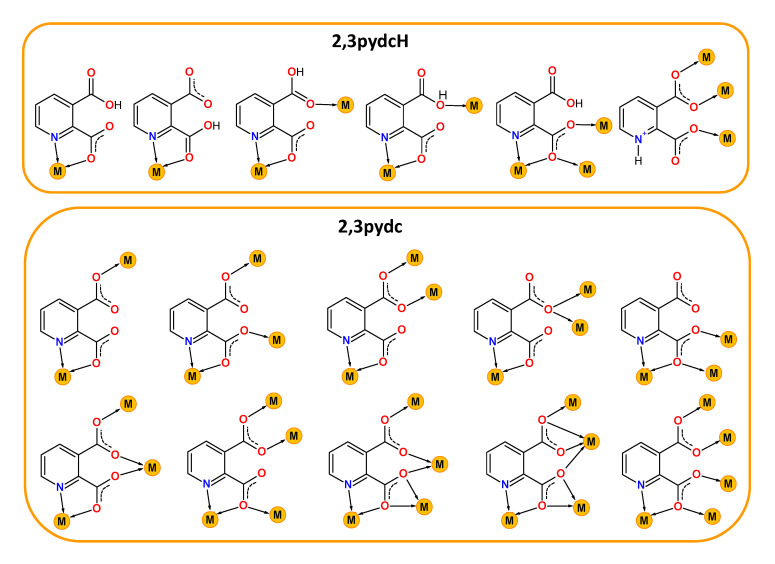
Selected coordination modes of mono-deprotonated-2,3pydcH (top) and deprotonated-2,3pydc (bottom) forms of pyridine-2,3-dicarboxylic acid (2,3pydcH_2_) in coordination with different metal ions reported in the literature [43,44,45,46,47,48,49,50,51,52,53,54,55].

Contemporary coordination chemistry establishes a scientific basis for obtaining compounds used as sources for synthesizing functional materials not only for technology [1,2,3,4,5,6,7] but also for medicine or pharmacy [8,9]. It is a great challenge to obtain proper functional materials because the properties of coordination compounds depend on the type of both metal ions and connecting ligands. Our interest in this field concerns the chemistry of lead(II) [10,11,12,13,14] and bismuth(III) coordination polymers with heteroaromatic carboxylate ligands. When selecting a central ion, we focused on the multiple applications of Bi(III) compounds as, for example, photoluminescent materials [15,16,17], catalysts [18], medical drugs for gastrointestinal disorders caused by *Helicobacter pylori* or anticancer agents [19,20,21,22]. Furthermore, the Bi(III) ion exhibits a [Xe]4*f*^14^5*d*^10^6*s*^2^ ground state electronic configuration and belongs to a group of metal ions (Tl(I), Pb(II), Sn(II), Sb(III)) possessing a lone electron pair (n*s*^2^). The presence of a lone electron pair, its stereochemical role and a relatively large bismuth covalent radius (1.48 Å [23]) can lead to the formation of interesting molecular structures of Bi-based complexes, including high coordination numbers and a hemi- or holodirected distribution of ligands around the metal center. When the lone electron pair is stereochemically inactive, it does not affect the geometry of the metal coordination environment. The bonds between metal and ligands are distributed symmetrically and the geometry around the central ion is defined as holodirected. While when the electron pair is stereochemically active, it occurs in a separate place in the central ion environment. The coordination bonds are distributed asymmetrically and only in a certain part of the coordination sphere. Such a type of geometry is called hemidirected [24]. These features make Bi(III) a coordinatively flexible ion, which is why we chose it for the synthesis of novel bismuth(III) coordination polymers. Additionally, the varied coordination structures of bismuth(III) complexes are also affected by the kind of donating ligand and the different coordination modes. This is especially important in the synthesis of functional coordination polymers (CPs), where ligands play an additional role as organic linkers. Among these linkers, carboxylate ligands are special, mainly due to the large variation in their coordination behavior. They can act as (i) monodentate κO, (ii) chelating κ^2^O,O′, (iii) bridging *µ*-κO:κO′, *µ*-κO:κO, *µ*_3_-κO:κO′:κO′, *µ*_4_-κO:κO:κO′:κO′ and (iv) chelating-bridging *µ*-κ^2^O,O′:κO′, *µ*_3_-κO:κ^2^O,O′:κO′, *µ*_4_-κO:κ^2^O,O′:κO′:κO′, *µ*_5_-κO:κO:κ^2^O,O′:κO′:κO′ ligands [25]. When researching examples of functional bismuth(III) coordination polymers in the literature, we focused on complexes with isomeric pyridine-dicarboxylic acids as linkers: pyridine-2,3-dicarboxylic acid (2,3pydcH_2_) [26], pyridine-2,5-dicarboxylic acid (2,5pydcH_2_) [27,28,29,30,31], pyridine-2,6-dicarboxylic acid (2,6pydcH_2_) [32,33,34,35,36,37], pyridine-3,4-dicarboxylic acid (3,4pydcH_2_) [37] and pyridine-3,5-dicarboxylic acid (3,5pydcH_2_) [38]. A literature search of coordination polymer functionality revealed six examples of Bi(III) complexes with luminescence properties [27,28,31,32,38]; three compounds exhibited gas-sorption behavior (CO_2_, N_2_, H_2_) [32], and one was used as a precursor for the preparation of Bi_2_O_3_ nanoparticles via thermal decomposition [26]. However, the biological activity of the mentioned complexes have not been examined, although all of the bismuth compounds clinically used against *Helicobacter pylori* belong to a group of Bi(III) carboxylates (bismuth subsalicylate (BSS, Pepto-Bismol), colloidal bismuth subcitrate (CBS, De-Nol) or ranitidine bismuth citrate (RBC, Pylorid)) [39,40]. Currently, the problems associated with this microaerophilic and neutralophilic Gram negative bacterium have been intensively investigated because it has been linked not only to many gastrointestinal disorders but also to gastric cancer. It should be noted that bismuth medications have been used not only against *H. pylori* as a component of quadruple therapies [41] as the first-line treatment (two antibiotics, proton-pump inhibitor and Bi-based drug), but Bi(III) complexes have also demonstrated anticancer activity. Additionally, bismuth was employed in radiation therapy in the form of Bi-nanoparticles and nanodots or in targeted radioimmunotherapy in the form of bismuth radionuclides [42]. Considering all of the above aspects of interesting bismuth chemistry, herein, we present the synthesis and characterization of new Bi(III) metal-organic coordination polymers. Taking into consideration our previous experience in the construction of CPs based on Pb(II) ions [10,11,12,13,14], we paid special attention to the choice of a proper organic linker. We selected pyridine-2,3-dicarboxylic acid as the prolinker for this purpose. It belongs to the group of multidonating N,O-donor ligands, which can exist in a mono- or a fully deprotonated form, indicating the possibility of different coordination modes with metal ions [43,44,45,46,47,48,49,50,51,52,53,54,55] (Scheme 1).

During the course of this study, we (i) synthesized and fully physicochemically characterized two novel bismuth(III) coordination polymers with pyridine-2,3-dicarboxylic acid (2,3pydcH_2_) as a prolinker; (ii) presented the molecular structure and topology of [Bi_2_(2,3pydc)_2_(2,3pydcH)_2_(H_2_O)]_n_ (**1**) and {(Et_3_NH)_2_[Bi(2,3pydc)(2,3pydcH)Cl_2_]}_n_ (**2**); (iii) confirmed and visualized the intermolecular close contacts in the crystal structures; (iv) carried out DFT (density functional theory) calculations to provide an explanation for the lone pair activity; (v) studied the luminescence properties of the obtained coordination polymers in the solid-state; and (vi) determined the bacteriostatic activity against *H. pylori,* which was compared with other Bi(III) carboxylate complexes.

## 2. Results and Discussion

### 2.1. Molecular Structure and Lone Pair Stereoactivity

**Figure 1:**
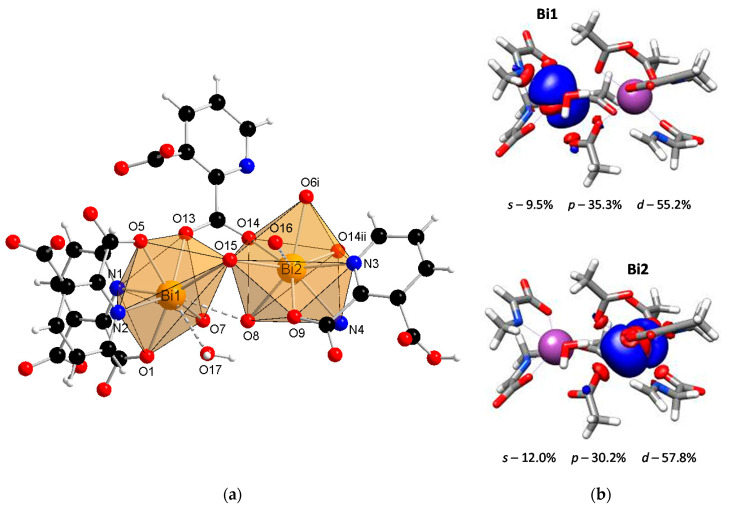
(**a**) The molecular structure with an atom numbering scheme of complex **1** (Symmetry codes: (i) −x, 1 − y, 1 − z; (ii) 1 − x, 1 − y, 1 − z) (Bi—orange, O—red, N—blue, C—black, H—white); (**b**) graphical representation of selected molecular orbital localized on Bi atom in complex **1** (DFT calculations).

The SC-XRD studies revealed that the coordination polymer [Bi_2_(2,3pydc)_2_(2,3pydcH)_2_(H_2_O)]_n_ (**1**) crystallized in the triclinic space group *P*-1. The asymmetric unit contained dimeric units of two crystallographically distinguishable Bi(III) centers. There were two anions of pyridine-2,3-dicarboxylic acid for each Bi center, one deprotonated (2,3pydc) and one mono-deprotonated (2,3pydcH). Additionally, there was one water molecule coordinated to the Bi1 center (Figure 1a). Each metal center existed in a different coordination environment. Around the Bi1 atom, there was a structural gap between the N2, O7 and O15 donor atoms, suggesting the influence of the lone electron pair on the geometry of the coordination sphere. The coordination bonds with those atoms were significantly longer (2.521–2.710 Å) than those with the other atoms (O5, O1 and N1, 2.314–2.386 Å) and were located opposite to the location of the lone electron pair (Table 1). Taking into consideration only the mentioned strong bonds, the geometry of the nearest coordination environment of the Bi1 center exhibited a coordination number of 7 and could be described as hemidirected. However, due to the significant stereoactivity of the lone electron pair, there were two additional donor atoms (O8 and O17) which were more distant from the Bi1 center (2.829 and 2.841 Å, respectively). These atoms, connected by weaker secondary bonds, completed the Bi1 coordination sphere. Therefore, the coordination number increased to 9, and the Bi1 coordination environment resembled a holodirected geometry [24]. The difference between the longest and shortest coordination bonds was 0.527 Å, which indicated a significant distortion of the coordination polyhedron. The Bi2 center was surrounded by eight donor atoms connected by strong primary bonds (2.311–2.650 Å) and one atom (O16) connected at a distance of 2.842 Å (secondary bond). In this case, the geometry of the Bi2 center was determined as hemidirected for CN = 8 or holodirected for CN = 9 [24].

It is important to confirm the conclusions drawn from the crystallographic analysis by DFT calculations. One of the crucial factors affecting the lone electron pair stereoactivity is the type of orbital, i.e., a spherical *s* orbital or space-oriented *p* or *d* orbitals, in which the electron density is mainly localized. Concerning compound **1**, the stereoactivity of the lone electron pair was slightly more pronounced in the Bi1 center than in Bi2 (Figure 1b). The localization of the lone electron pair in the Bi1 center indicated a significant contribution of the *p* orbital (35.3%) and a smaller contribution of the *s* orbital (9.5%). This caused the electron density to occupy a hemisphere in close proximity to the metal center. As a result, there was a gap in the structure, and the surrounding Bi1 atom adopted a hemidirected geometry. The lone pair stereoactivity in the Bi1 center caused the distortion of the coordination environment but simultaneously allowed for the formation of two additional coordination bonds with atoms located at a distance >2.8 Å. Therefore, the coordination geometry was not a typical hemidirected geometry since including secondary bonds filled up the hemisphere, resulting in a holodirected geometry. In turn, there was a smaller contribution of the *p* orbital (30.2%) and a slightly greater contribution of the *s* orbital (12.0%) in the localization of the electron pair in the Bi2 center than in the Bi1 center. As a result, the electron density in the Bi2 center affected the distortion of the coordination environment to a lower extent, and there was only one atom connected with the metal center by a secondary bond. However, concerning only primary bonds, the geometry of Bi2 was hemidirected, while including secondary bonds resulted in a holodirected geometry.

**Figure 2:**
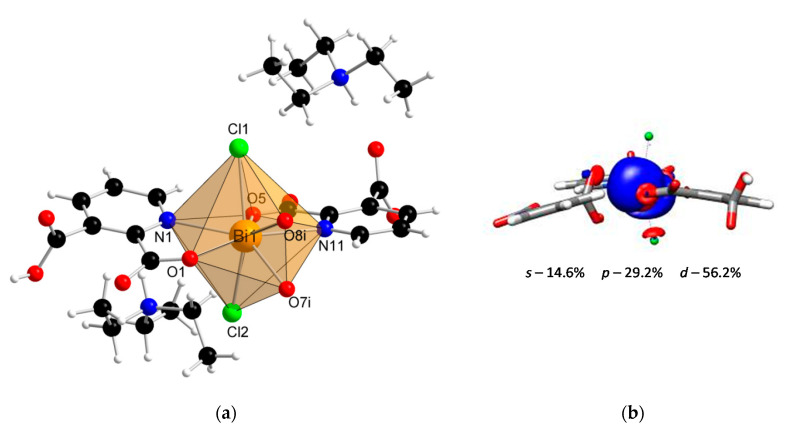
(**a**) The molecular structure with an atom numbering scheme of complex **2** (Symmetry code: (i) x, 0.5 − y, −0.5 + z) (Bi—orange, O—red, N—blue, Cl— green, C—black, H—white); (**b**) graphical representation of selected molecular orbital localized on Bi atom in complex **2** (DFT calculations).

Coordination polymer {(Et_3_NH)_2_[Bi(2,3pydc)(2,3pydcH)Cl_2_]}_n_ (**2**) crystallized in the monoclinic space group *P*2_1_/*c*. Its asymmetric unit comprised one Bi(III) ion, one molecule of pyridine-2,3-dicarboxylato ligand (2,3pydc), one molecule of 3-carboxypyridine-2-carboxylato ligand (2,3pydcH) and two coordinated chloride ions. Compound **2** is an anionic polymer, and the negative charge is neutralized by two triethylammonium cations for each Bi(III) center (Figure 2a). In contrast to complex **1**, the donor atoms in complex **2** were connected with the metal center only by primary bonds (CN = 8), which range from 2.374 to 2.725 Å (Table 2). The chromophore was formed as {BiO_4_N_2_Cl_2_}. The difference in the coordination bond lengths (0.351 Å) and angles slightly distorted the structure of the coordination environment from an ideal monocapped pentagonal bipyramid. However, a lack of obvious gaps and secondary bonds in the proximity of the Bi(III) center suggested that the lone electron pair was stereochemically inactive or weakly active and did not significantly affect the structure. Consequently, the nearest coordination environment of the Bi(III) center in compound **2** exhibited a holodirected geometry [24].

Additionally, the DFT calculations indicated the conclusion from structural studies. There was a smaller contribution of the *p* orbital (29.2%) and a slightly larger contribution of the *s* orbital (14.6%) to the localization of the lone electron pair in the Bi(III) center of compound **2** (Figure 2b) compared with the Bi(III) centers of compound **1**. As a result, the electron density was more centered and insignificantly influenced the structure distortion.

### 2.2. Topology and Supramolecular Structure

**Figure 3:**
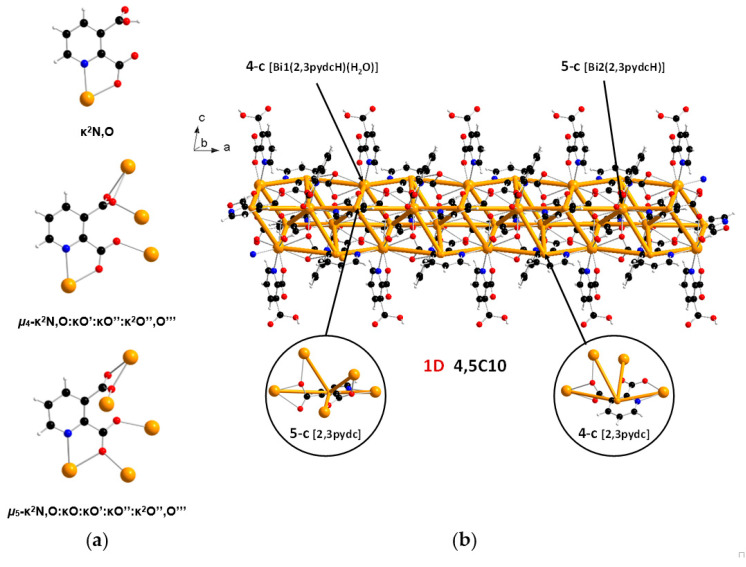
(**a**) Coordination modes of carboxylate ligands in complex **1**; (**b**) a fragment of the 1D crystal structure merged with **4,5C10** underlying net in valence-bonded MOFs standard representation for coordination polymer **1** (Bi—orange, O—red, N—blue, C—black, H—white).

In the structure of coordination polymer **1,** the monoanionic ligand molecules (2,3pydcH) were connected to the metal center via a chelating (κ^2^N,O) coordination. In turn, dianionic ligand molecules (2,3pydc) exhibit two kinds of chelating-bridging coordination modes. One of them connects four metal centers according to *µ*_4_-κ^2^N,O:κO′:κO″:κ^2^O″,O‴, while the second one bridges five Bi(III) centers according to *µ*_5_-κ^2^N,O:κO:κO′:κO″:κ^2^O″,O‴ (Figure 3a). The water molecule acted as a monocoordinated ligand. Bridging ligand molecules were responsible for the formation of 1D chains along the *a* axis with a **4,5C10** topology described by the (4^5^∙6)(4^9^∙6) point symbol. This type of the underlying net is constructed by 4-connected [Bi1(2,3pydcH)(H_2_O)] and 5-connected [Bi2(2,3pydcH)] nodes linked together by 4- and 5-connected [2,3pydc] linkers (Figure 3b). The Bi∙∙∙Bi distances ranged from 4.196 to 8.879 Å. The relatively short Bi∙∙∙Bi separations (about 4.2 Å) are close to the sum of the van der Waals radii (4.14 Å [56]). However, in the structure of **1** the shortest Bi∙∙∙Bi separations are those associated with Bi_2_O_2_ dimeric units. They might be considered simply a consequence of the bridging by O-donor carboxylate groups from 2,3pydc linkers and they are not indicative of a Bi∙∙∙Bi bond [57].

**Figure 4:**
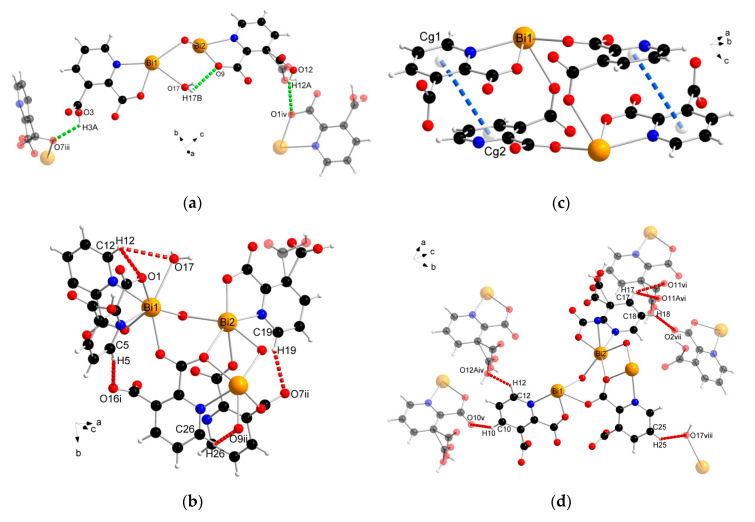
The geometry of noncovalent interactions in complex **1**: (**a**) O–H···O, (**b**) C–H···O intramolecular, (**c**) π···π and (**d**) C–H···O intermolecular (translucent molecules belong to adjacent chains) (Bi—orange, O—red, N—blue, C—black, H—white).

The supramolecular structure of coordination polymer **1** was stabilized through conventional hydrogen bonding (O–H∙∙∙O) between water molecules and the carboxylate ligands or between the carboxylic groups of the 2,3pydcH molecules and the carboxylate groups of the 2,3pydc ligands with *d*(H∙∙∙O) distances ranging from 1.801 to 2.574 Å (Figure 4a). Nonconventional (C–H∙∙∙O) hydrogen bonds, both intramolecular (Figure 4b) and intermolecular (Figure 4d), also played an important role. Moreover, π-stacking interactions between pyridine rings were observed in the structure at a distance of 4.043 Å (Figure 4c). The details of the interactions in complex **1** are listed in Table 3.

**Figure 5:**
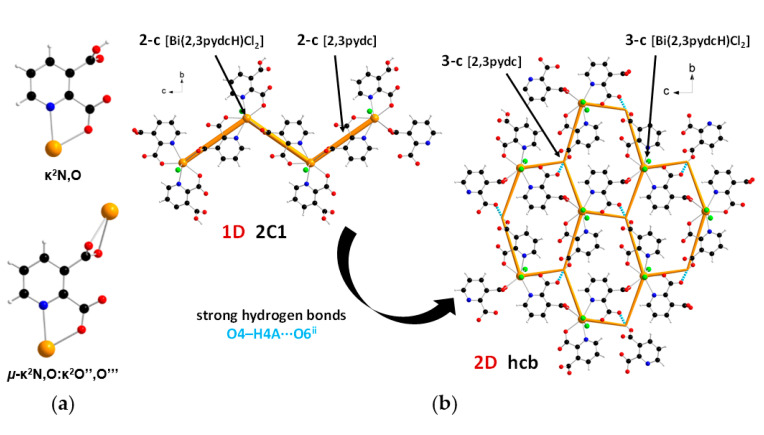
(**a**) Coordination modes of carboxylate ligands in complex **2**; (**b**) a fragment of the 1D crystal structure merged with **2C1** underlying net in valence-bonded MOFs standard representation and a fragment of the 2D crystal structure merged with **hcb** underlying the net in H-bonded MOFs standard representation for coordination polymer **2** (Bi—orange, O—red, N—blue, Cl—green, C—black, H—white).

In the structure of complex **2**, ligand 2,3pydcH exhibited a chelating coordination mode (κ^2^N,O), while ligand 2,3pydc exhibited a chelating-bridging coordination mode (*µ*-κ^2^N,O:κ^2^O″,O‴) (Figure 5a). Compound **2** was a 1-dimensional coordination polymer forming a zig-zag pattern along the *c* axis. From the topological point of view, this type of connection is described as **2C1** in the valence-bonded MOFs standard representation. 2-Connected [Bi(2,3pydcH)Cl_2_] nodes were combined by 2-connected [2,3pydc] linkers at a distance of 8.909 Å. In the H-bonded MOFs standard representation, the topology of complex **2** was changed. Taking into account the strong O4–H4A∙∙∙O6^ii^ hydrogen bonds formed between the –COOH group of the 2,3pydcH ligand and the –COO– group of the 2,3pydc ligand, the dimensionality increased to 2D (*bc* plane). Nodes and linkers became 3-connected, which resulted in the formation of a honeycomb-like **hcb** net described by the 6^3^ point symbol (Figure 5b).

**Figure 6:**
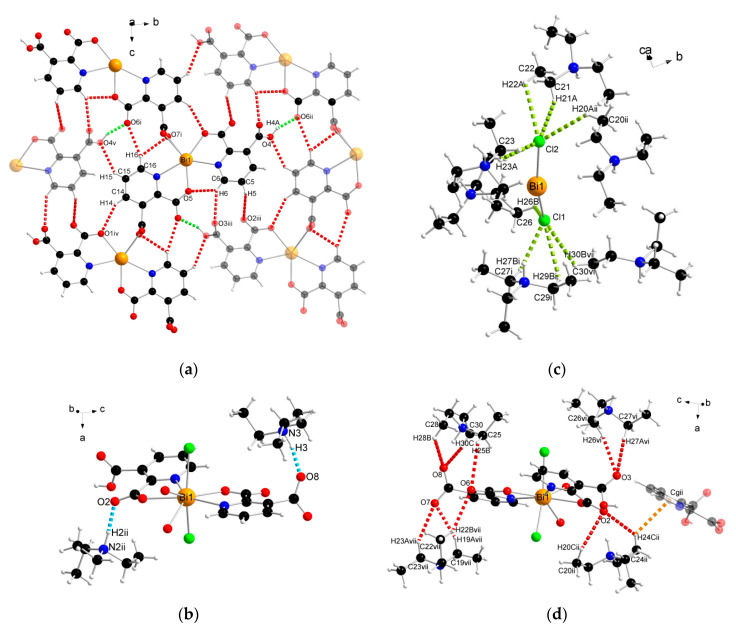
The geometry of noncovalent interactions in complex **2**: (**a**) O/C–H···O formed between adjacent polymeric chains, (**b**) N–H···O, (**c**) C–H···Cl and (**d**) C–H···O/π formed between Et_3_NH cations and polymeric chains (translucent molecules belong to adjacent chains) (Bi—orange, O—red, N—blue, Cl—green, C—black, H—white).

The analysis of the complex **2** crystal structure revealed many noncovalent interactions (Figure 6, Table 4). Between the 1D chains, strong O–H(COOH)∙∙∙O(COO) hydrogen bonds (H4A∙∙∙O6^ii^ = 1.727 Å) were formed (Figure 6a). Compound **2** was also stabilized by intra- and interchain C–H∙∙∙∙O hydrogen bonds with *d*(H∙∙∙O) distances ranging from 2.408 to 2.846 Å. The presence of triethylammonium cations in the structure was the source of many donor C–H and N–H groups, which were the basis of a strong hydrogen-bonded net. The stronger bonds were the N–H(Et_3_NH)···O(COO) bonds formed at *d*(H∙∙∙N) distances of 1.742 and 1.752 Å (Figure 6b). In turn, coordinated chloride ions served as acceptors in the formation of four-furcated C–H∙∙∙Cl hydrogen bonds with Et_3_NH cations (Figure 6c). Donor C–H groups of Et_3_NH molecules also formed interactions with carboxylate oxygen atoms at *d*(H∙∙∙O) distances of 2.554–2.999 Å (Figure 6d). Weak nonconventional C–H∙∙∙π hydrogen bond type III according to Malone [58] was also found (Figure 6d).

### 2.3. FT-IR Analysis

**Figure 7:**
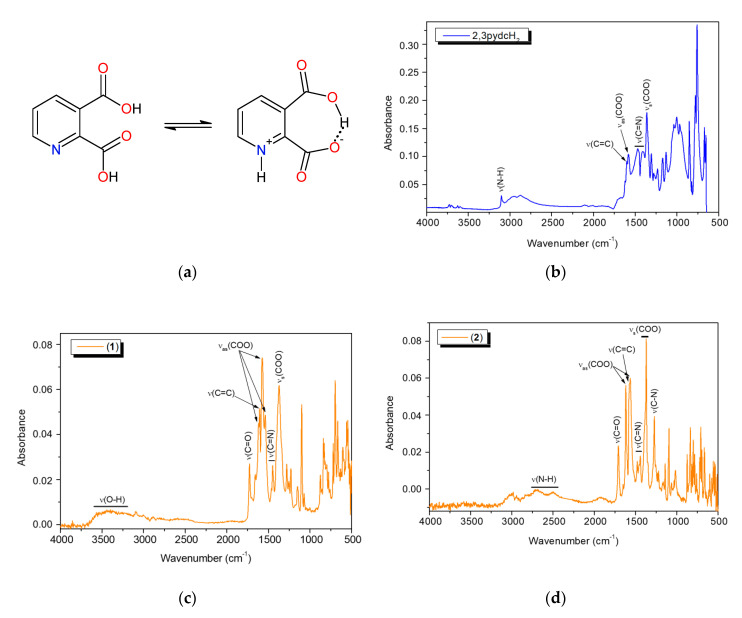
(**a**) The non-zwitterionic and zwitterionic form of 2,3pydcH_2_; the FT-IR spectra of: (**b**) 2,3pydcH_2_, (**c**) complex **1** and (**d**) complex **2**.

The FT-IR analysis results of pyridine-2,3-dicarboxylic acid and bismuth(III) complexes **1** and **2** are summarized in Table S1. An interesting phenomenon of this acid worth mentioning is that it exists in its zwitterionic form in the solid-state. Therefore, in the free ligand spectrum, we observed a band derived from protonated pyridine nitrogen (ν(N–H)_pyH_) and a broad band of O–H stretching vibrations attributed to intramolecular hydrogen bonds between the carboxylate groups [48,59,60]. The infrared spectra of bismuth coordination polymers **1** and **2** (Figure 7) contained the bands derived from the vibrations ν(C=O)_COOH_ at 1727 and 1708 cm^−1^, respectively, as well as the vibrations ν_as_(COO) and ν_s_(COO) (Table S1). This spectral pattern indicated that one of the carboxylic groups of the ligand was deprotonated while the second one remained deprotonated (mono-deprotonated-2,3pydcH anion), or both carboxylic groups were deprotonated (doubly deprotonated-2,3pydc anion). The Δν values of the carboxylate stretching frequencies [Δν = ν_as_(COO) − ν_s_(COO)] [61] were 246, 202 and 165 cm^−1^ in the spectrum of complex **1** and 249 and 182 cm^−1^ in the spectrum of complex **2,** indicating the different coordination modes of the carboxylates in these compounds. According to Deacon and Phillips [61], the value Δν > 200 cm^−1^ indicated a bridging mode of ligand binding, while Δν < 200 cm^−1^ indicated a bidentate chelating mode. In addition, the FT-IR spectra provided further evidence for the formation of a coordination bond between the Bi(III) ion and the pyridine nitrogen atom by means of a relative shift in the absorbance of the ν(C=N) band (see Table S1) and a strong absorption band at 634–624 cm^−1^ [35,62]. Additionally, in the spectrum of compound **1,** a wide band in the range 3600–3200 cm^−1^ from ν(O–H) vibrations corresponding to the water molecules engaged in hydrogen bonds was observed. On the other hand, in the spectrum of compound **2,** additional bands appeared at 2712 and 2511 cm^−1^ and at 1272 cm^−1^, which were attributed to the ν(N–H) and ν(C–N) vibrations. These bands suggested the presence of protonated triethylammonium (Et_3_NH) ions in the complex structure. Thus, spectroscopic analysis showed a good correlation with the structural X-ray analysis results of the analyzed complexes (vide supra).

### 2.4. Thermal Analysis

**Figure 8:**
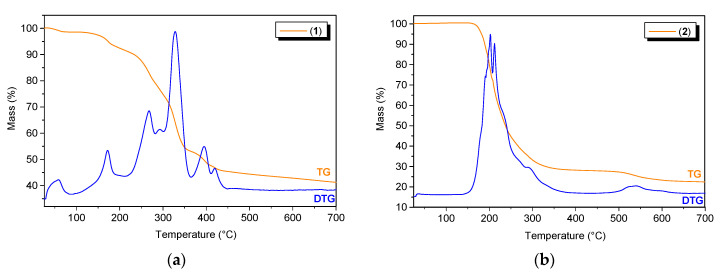
The TG/DTG curves for (**a**) compound **1** and (**b**) compound **2**.

To determine the thermal stability of the Bi(III) complexes, thermogravimetric analysis was carried out. Coordination polymer **1** was stable at temperatures up to 35 °C, and the first decomposition step was attributed to the loss of water molecules (1.59%, calcd: 1.64%) (Figure 8a). Next, at temperatures up to 450 °C, we observed the gradual decomposition of the coordinating mono-deprotonated and deprotonated pyridine-2,3-dicarboxylate anions (55.68%, calcd: 55.92%). At the end of this process, Bi_2_O_3_ remained as a final residue (42.73%, calcd: 42.44%). Polymer **2** was stable up to approximately 140 °C and then decomposed in two main stages (Figure 8b). The first stage involved the thermal dissociation of the two lattice triethylammonium cations (25.49%, calcd: 25.06%), which were bound to the coordination polymer chain by hydrogen bonds. In the next stage, which took place in the temperature range of 210–450 °C, the whole polymer was decomposed (observed: 46.29%, calcd: 46.37%). The product of the thermal decomposition of **2** was Bi_2_O_3_ (28.22%, calcd: 28.57%).

### 2.5. Photoluminescence Properties

**Figure 9:**
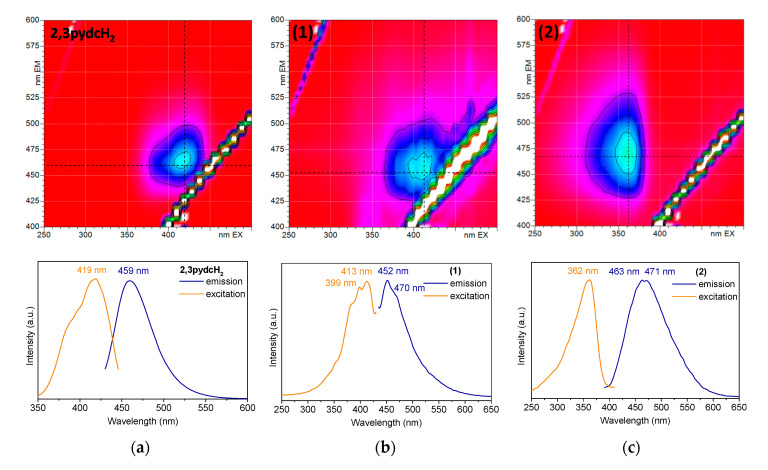
The solid-state 3D (top) and 2D (bottom) photoluminescence spectra of: (**a**) 2,3pydcH_2_, (**b**) compound **1** and (**c**) compound **2**.

To establish the photoluminescence properties of the newly obtained Bi(III) coordination polymers **1** and **2,** 3D and 2D room temperature solid-state excitation/emission spectra were recorded (Figure 9). Pyridine-2,3-dicarboxylic acid exhibited blue light emission with a maximum at 459 nm after excitation at 419 nm (Figure 9a). The emission of the ligand was attributed to the intraligand (IL) n→π* transitions [63]. The excitation spectrum of 2,3pydcH_2_ contained an additional band without a distinct maximum below 400 nm, which was attributed to the higher energy π→π* transitions. Comparing the 3D spectrum of compound **1** (Figure 9b) with the 2,3pydcH_2_ spectrum, the emission centers were located at similar wavelengths. The emission maximum at 452 nm (λ_ex_ = 413 nm) was only slightly blue-shifted (7 nm) in the spectrum of complex **1** compared to the ligand. The emission band was characterized by a similar ligand Stokes shift (39 nm), which pointed to the intraligand type of electronic transitions in complex **1**. The shoulder at ca. 470 nm indicated the presence of two types of ligand in the structure (mono-deprotonated and fully deprotonated), which coordinated to Bi(III) ions in different fashions. Thus, constructed in this way, the polymeric structure is characterized by a different rigidity, which influences the energy of emission. Similarly, compound **2** exhibited blue luminescence with a maximum at 463 and 471 nm after excitation at 362 nm (Figure 9c). Moreover, a significant Stokes shifts (101 and 109 nm) of coordination polymer **2**, accompanying by similar excitation wavelength compared to the ligand, suggested that the emission of **2** can be attributed to a ligand-to-metal charge transfer transition (LMCT), as well as to a change in the intraligand transitions (IL) [27,32,64]. The photoluminescence quantum yield of complex **2** was 8.36%, which makes it a good candidate for more specific studies of Bi-based fluorescent materials [62,65].

### 2.6. Solubility and Stability

Solubility and stability studies are important in the process of developing biologically active compounds. The ligand 2,3pydcH_2_ is soluble in H_2_O, 1 M HCl, MeOH, EtOH, DMF and DMSO, partly soluble in ^i^PrOH and MeCN, and insoluble in Me_2_CO, THF and CH_2_Cl_2_. However, coordination polymers **1** and **2** were soluble only in DMSO and 1 M HCl but insoluble in the other tested solvents. An important advantage of the tested bismuth compounds was their solubility in DMSO without subsequent precipitation after diluting it with water. It is an essential feature of compounds in further biological studies conducted against *Helicobacter pylori*.

**Figure 10:**
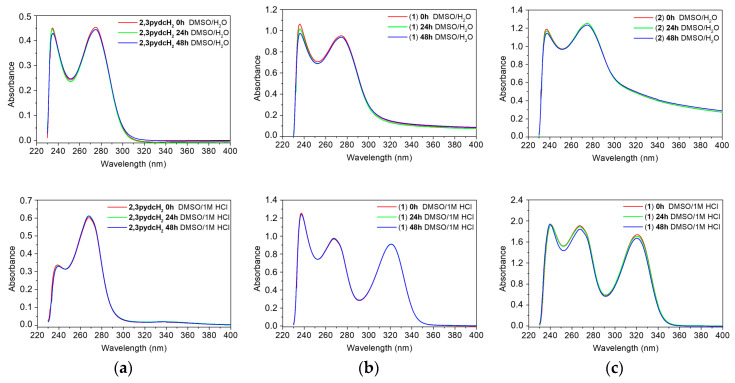
Time-dependent (0 h, 24 h and 48 h) stability studies on: (**a**) 2,3pydcH_2_, (**b**) complex **1**, (**c**) complex **2** in DMSO/H_2_O 1:50 (*v*/*v*) (top) and DMSO/1M HCl 1:50 (*v/v*) (bottom) solutions monitored by UV-Vis absorption spectra.

It is also important to examine the stability of analyzed compounds in the media used in the anti-*H. pylori* experiments. All tested compounds (2,3pydcH_2_, complexes **1** and **2**) were stable for 48 h when dissolved in DMSO and then diluted with H_2_O (1:50 *v/v*). According to the time-dependent UV-Vis spectra (Figure 10 (top)), there were no obvious changes in the positions of the absorption bands (±1 nm) and no significant decrease in absorbance. The absorption maxima were located at 236 and 274 nm for complex **1** and at 237 and 275 nm for complex **2** (Figure 10b,c (top)); the maxima were located almost at the same positions as the maxima in the ligand spectrum (234 and 275 nm) (Figure 10a (top)). Comparing the free ligand spectrum with the electronic spectra of complexes **1** and **2** showed the main bands corresponding to the ligand centered in the π→π* and n→π* transitions. Therefore, it can be assumed that there were no structural changes of the compounds upon their reaction with solvent (DMSO and H_2_O) molecules.

Additionally, it is important to examine the behavior of complexes **1** and **2** in acidic medium, taking into consideration that the compounds would be exposed to stomach acid in vivo if used as drugs [66]. After dissolving the compounds in 1 M HCl solution, the UV-vis spectra showed an additional absorption band near 321 nm, which confirmed that the complexes released organic acids and Bi^3+^ ions, which remained soluble in HCl solution as BiOCl [67]. A similar UV-Vis spectrum was recorded for the model reaction of BiCl_3_ with H_2_O in a 1 M HCl solution (Figure S1).

### 2.7. Antibacterial Activity towards Helicobacter Pylori

Infection with *H. pylori* bacteria contributes to the development of three serious digestive diseases, namely, gastric and duodenal ulcers, gastric cancer and MALT-type gastric lymphoma. The discovery of this relationship has led to extensive research regarding the application of bismuth compounds in the treatment of microbial infections [21,40]. The most important research studies include Bi(III) complexes with non-steroidal anti-inflammatory drugs [68], indolocarboxylates [67], salicylates [69,70,71], sulfonates [72,73,74], (thio)saccharinates [75], acetosulfame and cyclamic acid [76] and (benzo)hydroxamic acids [77,78]. Most of the compounds from these groups are heteroleptic organometallic complexes, including oxoclasters. A great advantage of the use of bismuth-based drugs is the lack of *H. pylori* resistance towards these drugs [22,41,79,80,81]. These studies inspired us to examine the antimicrobial properties of new polymeric Bi(III) complexes with low-molecular weight N-heteroaromatic carboxylic acids against *Helicobacter pylori*.

**Figure 11:**
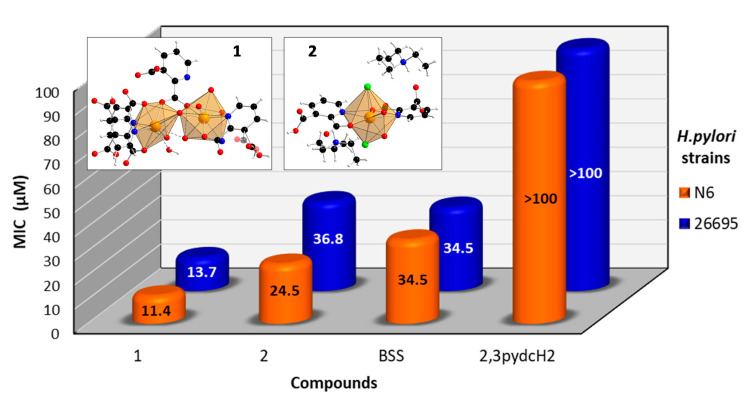
Antibacterial activity of [Bi_2_(2,3pydc)_2_(2,3pydcH)_2_(H_2_O)]_n_ (**1**), {(Et_3_NH)_2_[Bi(2,3pydc)(2,3pydcH)Cl_2_]}_n_ (**2**), pyridine-2,3-dicarboxylic acid (2,3pydcH_2_) and bismuth subsalicylate (BSS) against *H. pylori* strains 26695 and N6.

An analysis of the obtained results (Figure 11) revealed that the ligand 2,3pydcH_2_ was inactive against both *H. pylori* strains (26695 and N6), even at high concentrations (MIC > 100 μM). Both Bi(III) complexes exhibited bacteriostatic effects towards *H. pylori*. The MIC values of **1** and **2** towards reference strain 26695 reached values of 13.7 and 36.8 μM, respectively. The results indicated that the compound **1** was about twice as potent against *H. pylori* than the commercially used Bi-based drug BSS (MIC = 34.5 μM). Moreover, strain N6 was slightly more sensitive to compound **1** (MIC = 11.4 μM) and compound **2** (MIC = 24.5 μM) than strain 26695. Generally, compound **1** was more than twice as active against *H. pylori* as compound **2**. It can be concluded that the presence of chloride and triethylammonium ions in the structure of complex **2** pointed out the anionic form of Bi(III) complex, which influenced its antibacterial activity. The anionic form of complex **2** is more difficult to penetrate the negatively charged cell membrane of the *H. pylori* bacteria.

Comparing the antimicrobial activity of **1** and **2** with literature data for Bi(III)-carboxylate complexes, it was concluded that the obtained compounds exhibited a slightly smaller bacteriostatic effect towards *H. pylori* (Table 5). The reason for the lower activity could be explained by taking into consideration the polymeric form of the complexes and the different coordination environments, including not only oxygen but also nitrogen/chloride donor atoms and much more complicated forms of coordination polyhedra.

## 3. Experimental

### 3.1. Materials and Physicochemical Measurements

Chemical reagents were purchased commercially and used as received without further purification; Bi(NO_3_)_3_∙5H_2_O (POCH S.A., Gliwice, Poland), BiCl_3_ (Sigma-Aldrich, Steinheim, Germany), pyridine-2,3-dicarboxylic acid (Sigma-Aldrich, Steinheim, Germany), triethylamine (POCH S.A., Gliwice, Poland) and all organic solvents (Chempur, Piekary Śląskie, Poland). Elemental analysis (CHNS) was performed on an Elementar Vario Micro Cube analyzer (Elementar, Langenselbold, Germany). FT-IR spectra were recorded on a Nicolet 380 FT-IR type spectrophotometer (Thermo Scientific, Waltham, MA, USA), in the spectral range 4000–500 cm^−1^, using the ATR-diffusive reflection method. The thermal analysis (TG/DTG) was carried out using a TG/SDTA 851^e^ Mettler-Toledo thermobalance (Columbus, OH, USA). The experiments were performed in air atmosphere, at a heating rate of 5 °C min^−1^, in the temperature range of 25–700 °C, using α-Al_2_O_3_ crucible.

### 3.2. Synthesis of [Bi_2_(2,3pydc)_2_(2,3pydcH)_2_(H_2_O)]_n_ (***1***)

**Scheme 2:**
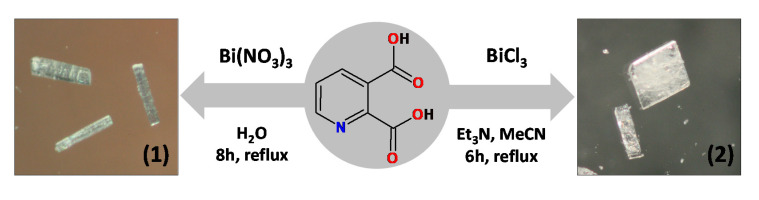
General reaction conditions employed in the synthesis of Bi(III) coordination polymers (**1** and **2**).

An aqueous solution (30 mL) of pyridine-2,3-dicarboxylic acid (0.75 mmol, 0.1253 g) was heated to about 100 °C. Next solid bismuth(III) nitrate (0.25 mmol, 0.1213 g) was added to the boiling solution. The resulting mixture was stirred and heated under a reflux for 8 h, after which it was filtered off. The clear filtrate was left to crystallize slowly at room temperature. After a week, colourless crystals were formed, collected by filtration and dried under vacuum (m = 0.0321 g (0.0292 mmol), yield: 23%, based on Bi(III) salt) (Scheme 2). Elemental analysis calculated for C_28_H_16_N_4_O_17_Bi_2_ (M_r_ = 1098.41) (%): C 30.62, H 1.47, N 5.10; found: C 30.43, H 1.40, N 5.17; FT-IR (cm^−1^): 3600–3200 (vb), 2887 (w), 1727 (m), 1657 (m), 1618 (m), 1604 (m), 1574 (s), 1537 (m), 1449 (w), 1372 (s), 1280 (m), 1254 (w), 1230 (m), 1149 (w), 1099 (s), 1071 (w), 1003 (w), 875 (w), 834 (m), 819 (m), 798 (w), 778 (w), 697 (s), 667 (m), 604 (m), 560 (m), 522 (m), 507 (w).

### 3.3. Synthesis of {(Et_3_NH)_2_[Bi(2,3pydc)(2,3pydcH)Cl_2_]}_n_ (***2***)

An acetonitrile solution (10 mL) of triethylamine (0.75 mmol, 0.0759 g) was added to 40 mL of acetonitrile solution of pyridine-2,3-dicarboxylic acid (0.75 mmol, 0.1253 g). To the boiling solution was added solid bismuth(III) chloride (0.25 mmol, 0.0788 g). The obtained mixture was heated for next 6 h under a reflux. The resulting mixture was filtered off and left at room temperature to evaporate slowly. After a week, colourless crystals were collected by filtration and dried under vacuum (m = 0.04479 g (0.0549 mmol), yield: 22%, based on Bi(III) salt) (Scheme 2). Elemental analysis calculated for C_26_H_39_N_4_O_8_Cl_2_Bi (M_r_ = 815.49) (%): C 38.29, H 4.82, N 6.87; found: C 38.14, H 4.65, N 6.78; FT-IR (cm^−1^): 2984 (w), 2712 (w), 2511 (b), 17708 (m), 1651 (w), 1618 (s), 1573 (s), 1563 (s), 1479 (w), 1445 (w), 1441 (m), 1395 (m), 1381 (m), 1370 (s), 1350 (w), 1272 (s), 1221 (w), 1140 (m), 1094 (m), 1063 (w), 1015 (w), 873 (m), 834 (m), 800 (m), 782 (w), 749 (m), 694 (w), 660 (m), 611 (w), 557 (w), 540 (w), 517 (w).

### 3.4. Crystal Data Collection and Refinement

Diffraction data were collected on an IPDS 2T dual-beam diffractometer (STOE & Cie GmbH, Darmstadt, Germany) at 120.0(2) K with Mo Kα radiation of the microfocus X-ray source (GeniX 3D Mo High Flux, Xenocs, 50 kV, 1.0 mA, λ= 0.71069 Å). The crystal was thermostated in a nitrogen stream at 120 K using the CryoStream-800 device (Oxford CryoSystem, Long Hanborough, UK) during the entire experiment. Data collection and data reduction were controlled by the X-Area 1.75 program (STOE) [82]. Owing to the high absorption coefficients (11.345 and 5.742 mm^−1^), numerical absorption corrections were applied based on measured crystal faces. The structures of **1** and **2** were solved with the ShelXT [83] structure solution programs run under Olex2 [84] using Intrinsic Phasing and refined with the ShelXL [85] refinement package. The WinGX [86] program was used to prepare the final version of CIF files. Diamond [87] was used to prepare the figures. All C–H type hydrogen atoms were attached at their geometrically expected positions and refined as riding on heavier atoms with the usual constraints. Positions of the N–H and O–H hydrogen atoms were calculated geometrically and taken into account with isotropic temperature factors and refined as constrained, using the AFIX 13 (for N–H), AFIX 6 and AFIX 147 (for O–H) instructions. Aromatic ring (N3, C15–C19) together with the carboxylate group C(21)O(11)O(12)H(12a) in **1** was found disordered in two positions with probabilities of 0.60(2) and 0.40(2). One atom Cl2 and five ethyl groups in **2** have been modelled as disordered: Cl2 (s.o.f. 0.69(13)/0.31(13)); C19–C20 (s.o.f. 0.71(4)/0.29(4)); C21–C22 (s.o.f. 0.52(8)/0.48(8)); C23–C24 (s.o.f. 0.86(3)/0.14(3)); C25–C26 (s.o.f. 0.51(4)/0.49(4)); C29–C30 (s.o.f. 0.63(5)/0.37(5)). In compounds **1** and **2**, residual electron density is somewhat high and localizes near the heavier Bi atom. In our opinion it is an artefact (e.g., due to cut of Fourier series), since no atom can be present at that location. A summary of crystallographic data is shown in Table 6. Crystallographic data for structure of **1** and **2** reported in this paper have been deposited with the Cambridge Crystallographic Data Centre as supplementary publications No. CCDC 1911680 and 1911679. Copies of the data can be obtained free of charge on application to CCDC, 12 Union Road, Cambridge CB2 1EZ, UK (Fax: (+44)-1223-336-033; E-mail: deposit@ccdc.cam.ac.uk).

### 3.5. Topological Analysis

Topological analysis of the metal-organic network of coordination polymers obtained was performed with the ToposPro program package [88] and the TTD collection of periodic network topologies following the concept of the simplified underlying net. Such a net was generated by contracting organic ligands to their centroids, maintaining their connectivity via coordination bonds and including secondary bonds. The underlying networks obtained have been topologically analyzed and classified [89,90].

### 3.6. DFT Calculations

Quantum-chemical calculations were performed in the framework of density-functional theory (DFT). Hybrid three-parameter Becke Lee-Yang-Parr functional B3LYP [91] together with Ahlrichs’ triple-zeta split-valence basis set was augmented by Coulomb fitting (def2-TZVP) [92] using resolution of the identity approximation [93]. The Stuttgart-Dresden effective core potential (ECP) for 60 core electrons of Bi was used [94]. The ORCA ab initio, DFT and semiempirical SCF-MO package [95] was used for all calculations. Truncated parts of [Bi_2_(2,3pydc)_6_(2,3pydcH)_2_(H_2_O)]^8−^ (**1**) and [Bi(2,3pydc)_2_(2,3pydcH)Cl_2_]^4−^ (**2**) were used as models for the DFT calculations. The model of **1** appeared too large for calculations, so the ligands were truncated, keeping the positions of N,O-donor atoms and some of non-coordinative organic fragments unchanged in comparison to the initial structure. In the final case, the fragment of [Bi_2_(AcO)_4_(H_2_O)(CH_2_NCH_2_COO)(CH_3_NC(CH_3_)COO)_2_(CH_3_NCHCOO)] for **1** was included in the calculations. The positions of hydrogen atoms were optimized, while other atoms retained the same coordinates as determined from single-crystal X-ray analysis (*cif* files). Molecular orbitals were localized for analysis using Foster-Boys (spatial localization) algorithm [96]. Electron density distributions were visualized with the UCSF Chimera package [97].

### 3.7. Solid-State Photoluminescence Measurements

The solid-state photoluminescence spectra were recorded at room temperature on a spectrofluorophotometer RF-5301 equipped with a 150 W Xenon lamp and a solid sample holder (Shimadzu, Kioto, Japan). The 3D emission spectra and 2D excitation and emission spectra were collected with a 1.5 nm slit width for excitation and a 3 nm slit width for emission monochromators. Photoluminescence data were processed using Panorama (LabCognition GMBH, Cologne, Germany) software. Photoluminescence quantum yield was measured on a FLS980 spectrofluorimeter (Edinburgh Instruments, Livingston, UK), equipped with an integrating sphere, using BaSO_4_ as a reference.

### 3.8. Solubility and Stability Studies

Solubility studies were performed using H_2_O, 1M HCl and commonly used organic solvents: MeOH, EtOH, ^i^PrOH, MeCN, DMF, DMSO, Me_2_CO, THF and CH_2_Cl_2_. UV-Vis stability measurements were conducted on a V-630 Jasco UV-Vis spectrophotometer (Jasco Corporation, Tokyo, Japan) using 1 cm cuvettes. Analyzed compounds (ligand 2,3pydcH_2_, complexes **1** and **2**) were dissolved in DMSO and diluted with H_2_O or 1M HCl 1:50 (*v*/*v*) to give concentration of 10^−4^ M. All the time-dependent (0 h, 24 h and 48 h) measurements were recorded at room temperature. The UV-Vis spectrum of product of the reaction BiCl_3_ with H_2_O was measured in 1M HCl with an addition of 2% *v*/*v* DMSO at a concentration of 10^−4^ M at room temperature.

### 3.9. Bacterial Strains and Culture Conditions

Two *Helicobacter pylori* strains, 26695 [98] and N6 [99], were used to determine the Minimum Inhibitory Concentration (MIC) of the synthesized Bi-compounds by agar dilution. *H. pylori* 26695 and N6 strains were cultured on Columbia Blood Agar Base plates (CBA, CM331, Oxoid, Basingstoke, UK) supplemented with 10% (*v*/*v*) defibrinated horse blood. Liquid cultures were grown with shaking at 140 rpm in BBL Brucella Broth (BBL, 211088, Beckton Dickinson, Franklin Lakes, NJ, USA) with 10% foetal calf serum (Biowest S1810). Liquid and solid *H. pylori* cultures were supplemented with 0.2% (*v/v*) mixture of antibiotics containing 0.3 mg L^−1^ polymyxin B, 12.5 mg L^−1^ vancomycin, 2.5 mg L^−1^ amphotericin B and 6.25 mg L^−1^ trimethoprim [100]. *H. pylori* were cultivated under microaerobic conditions (5% O_2_, 10% CO_2_, 85% N_2_) at 37 °C.

### 3.10. Determination of The Minimum Inhibitory Concentration (MIC)

The MIC values of bismuth(III) complexes (**1** and **2**), ligand (pyridine-2,3-dicarboxylic acid) and bismuth subsalicylate (BSS, Sigma-Aldrich, Steinheim, Germany) as a reference were determined by the agar dilution technique. All compounds were dissolved in DMSO and mixed with CBA to give the desired concentrations of the tested compounds. In the initial screening, concentrations ranging from 1 to 100 µg mL^−1^ at intervals of 20 µg mL^−1^ were used. In the final analyses, the bismuth compound concentrations were used at intervals of 2.5 µg mL^−1^. *H. pylori* liquid cultures of OD_600_ ~0.5–1.5 (log-phase, OD_600_ = 1 corresponds to ~6.5 × 10^8^ CFU mL^−1^) were diluted in BBL to ~10^5^ CFU mL^−1^. Aliquots (100 µL) of bacterial suspensions were then streaked onto previously prepared CBA plates containing the different concentrations of tested compounds. CBA plates with DMSO at the same concentration as in CBA plates with compounds, or without DMSO, were used as controls of *H. pylori* growth under experimental conditions. The plates were incubated for 5 days, and then *H. pylori* growth was assessed. The lowest concentration of a compound at which no *H. pylori* growth was observed was considered as MIC.

## 4. Conclusions

In summary, we synthesized and fully physicochemically characterized two novel bismuth(III) coordination polymers, with pyridine-2,3-dicarboxylic acid (2,3pydcH_2_) as the prolinker. The molecular structures of the obtained coordination polymers [Bi_2_(2,3pydc)_2_(2,3pydcH)_2_(H_2_O)]_n_ (**1**) and {(Et_3_NH)_2_[Bi(2,3pydc)(2,3pydcH)Cl_2_]}_n_ (**2**) indicated that the coordination environments of the metal centers were different, mainly due to the influence of a free electron pair. The crystallographic studies and quantum-chemical calculations pointed to a hemidirected geometry of bismuth centers in **1** and a holodirected geometry in **2**. The various coordination modes of bridging carboxylate ligands (*µ*_4_-κ^2^N,O:κO′:κO″:κ^2^O″,O‴ and *µ*_5_-κ^2^N,O:κO:κO′:κO″:κ^2^O″,O‴ in **1** and *µ*-κ^2^N,O:κ^2^O″,O‴ in **2**) were responsible for the formation of 1D chains with **4,5C10** and **2C1** topologies. Both coordination polymers exhibited blue-light photoluminescence with emission maxima at 452 nm and 463/471 nm for **1** and **2**, respectively. The photoluminescence quantum yield of compound **2** was 8.36%, which makes it a good candidate for more specific studies of Bi-based fluorescent materials. Additionally, the in vitro tests evaluating the bacteriostatic activity against *Helicobacter pylori* showed that Bi(III) coordination polymers had good antibacterial properties. The MIC values of **1** and **2** towards reference strain 26695 reached values of 13.7 and 36.8 μM, respectively. It can be concluded that differences in the structures of coordination polymers ([Bi_2_(2,3pydc)_2_(2,3pydcH)_2_(H_2_O)]_n_ (**1**) and {(Et_3_NH)_2_[Bi(2,3pydc)(2,3pydcH)Cl_2_]}_n_ (**2**)) influences the antibacterial activity. Additionally, the *H. pylori* strain N6 was slightly more sensitive to compound **1** (MIC = 11.4 μM) than strain 26,695. Therefore, more detailed research in this field should be conducted.

## Figures and Tables

**Table 1:** Selected bond lengths (Å) and angles (°) for complex **1**.

Bond Lengths (Å)							
Bi1–O5	2.314(10)	Bi1–O7	2.697(9)	Bi2–O9	2.311(9)	Bi2–O14 ^ii^	2.539(9)
Bi1–O1	2.332(9)	Bi1–O15	2.710(9)	Bi2–O15	2.433(9)	Bi2–N4	2.640(11)
Bi1–N1	2.386(12)	Bi1–O8	2.829(10)	Bi2–O8	2.484(10)	Bi2–O14	2.650(10)
Bi1–O13	2.443(9)	Bi1–O17	2.841(10)	Bi2–O6 ^i^	2.491(9)	Bi2–O16	2.842(10)
Bi1–N2	2.521(12)			Bi2–N3	2.506(10)		
**Bond Angles (°)**							
O1–Bi1–N2	74.3(4)	O5–Bi1–O7		141.6(3)	O15–Bi2–N3		117.4(4)
O1–Bi1–N1	68.8(4)	O5–Bi1–O17		121.4(3)	O15–Bi2–N4		141.6(3)
O1–Bi1–O5	131.9(3)	O13–Bi1–O15		72.8(3)	O15–Bi2–O9		77.2(3)
O1–Bi1–O13	135.0(3)	O13–Bi1–O8		88.2(3)	O14–Bi2–O16		117.2(3)
O1–Bi1–O15	143.0(3)	O13–Bi1–O7		77.9(3)	O14–Bi2–O6 ^i^		70.4(3)
O1–Bi1–O8	88.8(3)	O13–Bi1–O17		140.9(3)	O14–Bi2–O14 ^ii^		71.6(3)
O1–Bi1–O7	67.3(3)	O15–Bi1–O8		65.0(3)	O14–Bi2–N3		145.4(3)
O1–Bi1–O17	71.9(3)	O15–Bi1–O7		106.3(3)	O14–Bi2–N4		111.2(3)
N2–Bi1–N1	84.0(4)	O15–Bi1–O17		72.6(3)	O14–Bi2–O9		144.6(3)
N2–Bi1–O5	66.1(3)	O8–Bi1–O7		47.9(3)	O16–Bi2–O6 ^i^		79.4(3)
N2–Bi1–O13	130.9(3)	O8–Bi1–O17		60.9(3)	O16–Bi2–O14 ^ii^		138.5(3)
N2–Bi1–O15	107.2(3)	O7–Bi1–O17		95.0(3)	O16–Bi2–N3		69.3(4)
N2–Bi1–O8	137.8(3)	O8–Bi2–O15		74.5(3)	O16–Bi2–N4		131.2(3)
N2–Bi1–O7	141.2(3)	O8–Bi2–O14		72.2(3)	O16–Bi2–O9		65.1(3)
N2–Bi1–O17	77.1(4)	O8–Bi2–O16		115.1(3)	O6 ^i^–Bi2–O14 ^ii^		65.4(3)
N1–Bi1–O5	80.6(4)	O8–Bi2–O6 ^i^		142.4(3)	O6 ^i^–Bi2–N4		78.3(4)
N1–Bi1–O13	77.0(4)	O8–Bi2–O14 ^ii^		106.2(3)	O6 ^i^–Bi2–N4		122.8(3)
N1–Bi1–O15	147.6(3)	O8–Bi2–N3		138.0(6)	O6 ^i^–Bi2–O9		138.6(3)
N1–Bi1–O8	126.0(4)	O8–Bi2–N4		75.4(3)	O14 ^ii^–Bi2–N3		82.5(5)
N1–Bi1–O7	78.1(4)	O8–Bi2–O9		75.5(3)	O14 ^ii^–Bi2–N4		61.7(3)
N1–Bi1–O17	139.7(4)	O15–Bi2–O14		80.7(3)	O14 ^ii^–Bi2–O9		132.0(3)
O5–Bi1–O13	66.4(3)	O15–Bi2–O16		48.6(3)	N3–Bi2–N4		73.3(6)
O5–Bi1–O15	76.8(3)	O15–Bi2–O6 ^i^		95.5(3)	N3–Bi2–O9		69.2(6)
O5–Bi1–O8	139.1(3)	O15–Bi2–O14 ^ii^		150.7(3)	N4–Bi2–O9		73.3(3)

Symmetry codes: ^i^ −x, 1 − y, 1 − z; ^ii^ 1 − x, 1 − y, 1 − z.

**Table 2:** Selected bond lengths (Å) and angles (°) for complex **2**.

Bond Lengths (Å)							
Bi1–O5	2.374(7)	Bi1–N1	2.546(7)	Bi1–O7 ^i^	2.651(6)	Bi1–O8 ^i^	2.721(7)
Bi1–O1	2.429(7)	Bi1–N11	2.585(7)	Bi1–Cl2	2.672(2)	Bi1–Cl1	2.725(3)
**Bond Angles (°)**							
O1–Bi1–N1		65.5(2)	N1–Bi1–N11		140.9(2)	O5–Bi1–O7 ^i^	136.5(2)
O1–Bi1–Cl1		97.3(3)	N1–Bi1–O7 ^i^		138.2(2)	O5–Bi1–Cl2	83.6(8)
O1–Bi1–O5		140.5(2)	N1–Bi1–Cl2		82.8(5)	O8 ^i^–Bi1–N11	81.3(2)
O1–Bi1–O8 ^i^		77.5(2)	Cl1–Bi1–O5		83.5(3)	O8 ^i^–Bi1–O7 ^i^	48.4(2)
O1–Bi1–N11		153.5(2)	Cl1–Bi1–O8 ^i^		74.3(2)	O8 ^i^–Bi1–Cl2	125.8(5)
O1–Bi1–O7 ^i^		75.2(2)	Cl1–Bi1–N11		92.0(2)	N11–Bi1–O7 ^i^	78.8(2)
O1–Bi1–Cl2		82.3(10)	Cl1–Bi1–O7 ^i^		122.7(2)	N11–Bi1–Cl2	97.6(9)
N1–Bi1–Cl1		77.8(2)	Cl1–Bi1–Cl2		158.8(3)	O7 ^i^–Bi1–Cl2	77.9(4)
N1–Bi1–O5		76.3(2)	O5–Bi1–O8 ^i^		138.8(2)		
N1–Bi1–O8 ^i^		129.7(2)	O5–Bi1–N11		65.1(2)		

Symmetry code: ^i^ x, 0.5 − y, −0.5 + z.

**Table 3:** The geometry of noncovalent interactions in complex **1**.

Hydrogen Bonds				
Donor–H···Acceptor	D–H (Å)	H···A (Å)	D···A (Å)	D–H···A (°)
O3–H3A···O7 ^iii^	0.84(1)	1.801(28)	2.625(37)	166.46(4)
O12–H12A···O1 ^iv^	0.84(1)	2.550(41)	3.171(40)	131.65(5)
O17–H17B···O9	0.87(1)	2.574(14)	3.208(30)	130.56(4)
C5–H5···O16 ^i^	0.95(1)	2.220(38)	3.140(51)	162.68(4)
C10–H10···O16 ^i^	0.95(1)	2.489(38)	3.310(47)	144.71(5)
C12–H12···O1	0.95(1)	2.542(41)	3.012(42)	110.73(4)
C12–H12···O17	0.95(1)	2.926(38)	3.409(51)	112.79(3)
C12–H12···O12 A ^iv^	0.95(1)	2.211(36)	2.957(53)	134.64(4)
C17–H17···O11 ^vi^	0.95(2)	2.519(5)	3.313(13)	141.00(3)
C17–H17···O11 A ^vi^	0.95(2)	2.699(41)	3.278(36)	119.81(3)
C18–H18···O2 ^vii^	0.95(2)	2.901(10)	3.496(23)	121.81(3)
C19–H19···O7 ^ii^	0.95(1)	2.483(20)	3.346(22)	150.44(6)
C25–H25···O17 ^viii^	0.95(1)	2.639(36)	3.336(38)	130.62(5)
C26–H26···O9 ^ii^	0.95(1)	2.467(36)	2.948(25)	111.30(4)
**π···π interactions**				
	**Cg···Cg (Å)**			
Cg1···Cg2	4.043(24)			

Symmetry codes: ^i^ −x, 1 − y, 1 − z; ^ii^ 1 − x, 1 − y, 1 − z; ^iii^ 1 − x, 1 − y, −z; ^iv^ 1 − x, −y, 1 − z; ^v^ −x, −y, 1 − z; ^vi^ 1 − x, −y, 2 − z; ^vii^ x, y, 1 + z; ^viii^ x, 1 + y, z. Cg denotes the centre of gravity of the 6-membered pyridine ring.

**Table 4:** The geometry of noncovalent interactions in complex **2**.

Hydrogen Bonds					
Donor–H···Acceptor		D–H (Å)	H···A (Å)	D···A (Å)	D–H···A (°)
Interchain	O4–H4A···O6 ^ii^	0.84	1.727(1)	2.533(1)	160.08
	C5–H5···O2 ^iii^	0.95	2.846(6)	3.281(6)	109.03
	C6–H6···O3 ^iii^	0.95	2.654(3)	3.460(3)	143.04
	C6–H6···O5	0.95	2.408(1)	3.025(1)	122.36
	C14–H14···O1 ^iv^	0.95	2.585(2)	3.399(2)	143.88
	C15–H15···O4 ^v^	0.95	2.545(2)	3.323(1)	139.22
	C16–H16···O6 ^i^	0.95	2.689(1)	3.598(1)	160.22
	C16–H16···O7 ^i^	0.95	2.661(2)	3.280(2)	123.17
Between Et_3_NH and polymeric chain	N2 ^ii^–H2 ^ii^···O2	1.00	1.742(1)	2.740(2)	174.95
	N3–H3···O8	1.00	1.752(2)	2.701(3)	156.94
	C26–H26B···Cl1	0.97	2.801(4)	3.523(5)	131.09
	C27 ^i^–H27B ^i^···Cl1	0.99	2.778(3)	3.507(3)	130.83
	C29 ^i^–H29B ^i^···Cl1	0.98	3.004(5)	3.696(5)	128.49
	C30 ^vi^–H30B ^vi^···Cl1	0.98	3.058(2)	3.745(2)	128.29
	C20 ^ii^–H20A ^ii^···Cl2	0.98	1.992(1)	2.458(3)	106.68
	C21–H21A···Cl2	0.99	2.925(1)	3.615(2)	127.11
	C22–H22A···Cl2	0.99	2.986(2)	3.609(4)	121.93
	C23–H23A···Cl2	0.99	3.052(1)	4.012(2)	163.96
	C19 ^vii^–H19A ^vii^···O7	0.99	2.554(4)	3.410(5)	144.9
	C20 ^ii^–H20C ^ii^···O2	0.98	2.986(5)	3.640(5)	125.41
	C22 ^vii^–H22B ^vii^···O6	0.98	2.766(3)	3.549(6)	137.45
	C23 ^vii^–H23A ^vii^···O7	0.99	2.624(4)	3.475(3)	144.18
	C24 ^ii^–H24C ^ii^···O2	0.98	2.840(3)	3.544(6)	129.43
	C25–H25B···O6	0.99	2.922(1)	3.396(2)	110.41
	C26 ^vi^–H26C ^vi^···O3	0.98	2.642(4)	3.610(6)	170.72
	C27 ^vi^–H27A ^vi^···O3	0.99	2.606(3)	3.594(4)	175.75
	C28–H28B···O8	0.98	2.999(3)	3.600(3)	120.91
	C30–H30C···O8	0.99	2.919(6)	3.640(8)	130.61
	C24–H24C···Cg ^ii^	0.98	2.967(5)	3.627(5)	125.63

Symmetry codes: ^i^ x, 0.5 − y, −0.5 + z; ^ii^ x, 1.5 − y, −0.5 + z; ^iii^ x, 1.5 − y, 0.5 + z; ^iv^ x, 0.5 − y, 0.5 + z; ^v^ x, −1 + y, z; ^vi^ −x, 0.5 + y, 1.5 − z; ^vii^ 1 − x, 1 − y, 2 − z. Cg denotes the centre of gravity of the 6-membered pyridine ring. Et_3_NH—triethylammonium cation.

**Table 5:** Antibacterial activity of Bi(III) carboxylate complexes against *Helicobacter pylori* strain 26695.

Compound	Representation of Bi(III) Coordination	Chromophore	MIC µg mL^−1^ (µM)	Ref.
*Pyridinedicarboxylic acid*				
[Bi_2_(2,3pydc)_2_(2,3pydcH)_2_(H_2_O)]*_n_* (**1**)	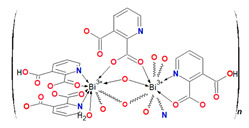	{BiO_7_N_2_}	15.0 (13.7)	This work
{(Et_3_NH)_2_[Bi(2,3pydc)(2,3pydcH)Cl_2_]}*_n_* (**2**)	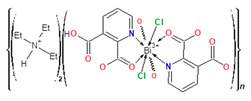	{BiO_4_N_2_Cl_2_}	30.0 (36.8)	
BSS			12.5 (34.5)	
*Indolecarboxylic acid*				
[Bi(IAA)_3_]	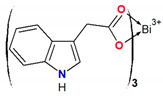 [Bi(IAA)_3_]	{BiO_6_}	6.25 (8.54)	[67]
[Bi(IPA)_3_]		{BiO_6_}	6.25 (8.07)	
[Bi(IBA)_3_]		{BiO_6_}	6.25 (7.66)	
[Bi(MICA)_3_]		{BiO_6_}	6.25 (8.54)	
[Bi(IGA)_3_]		{BiO_6_}	6.25 (8.08)	
BSS			12.5 (34.5)	
*Acetylsalicylic acid*				
[Bi(asp)_3_]_∞_	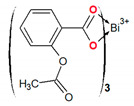 [Bi(asp)_3_]_∞_	{BiO_8_}	6.25 (8.37)	[70]
[KBi(asp)_4_]_∞_		{BiO_9_}	6.25 (6.48)	
*NSAIDs*				
[Bi(L^1^)_3_·H_2_O]	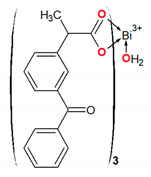 [Bi(Ketoprofen)_3_·H_2_O]	{BiO_6_}	≥6.25 (6.35)	[68]
			≥6.25 (4.85)	
[Bi(L^2^)_3_]		{BiO_6_}	≥6.25 (7.01)	
			≥6.25 (7.58)	
			≥6.25 (6.47)	
			≥6.25 (8.67)	
			≥6.25 (6.75)	
			≥6.25 (6.55)	
			≥6.25 (6.33)	
			≥6.25 (5.97)	
BSS			≥12.5 (34.5)	

2,3pydcH_2_—pyridine-2,3-dicarboxylic acid; Et_3_N—triethylamine; BSS—bismuth subsalicylate; IAA-H—2-(1*H*-indol-3-yl)acetic acid, IPA-H—3-(1*H*-indol-3-yl)propanoic acid, IBA-H—4-(1*H*-indol-3-yl))butanoic acid, IGA-H—2-(1*H*-indol-3-yl))-2-oxoacetic acid, MICA-H—1-methyl-1*H*-indole-3-carboxylic acid; aspH—acetylsalicylic acid; NSAIDs: L^1^: Ketoprofen, Sulindac; L^2^: Naproxen, Ibuprofen, Fenbufen, 5-Chlorosalicylic acid, Mefenamic acid, Diflunisal, Tolfenamic acid, Flufenamic acid.

**Table 6:** Crystal data and structure refinement for **1** and **2**.

Empirical Formula	C_28_H_16_Bi_2_N_4_O_17_	C_26_H_39_BiCl_2_N_4_O_8_
CCDC number	1911680	1911679
Formula weight (g mol^−1^)	1098.41	815.49
Temperature (K)	120(2)	120(2)
Wavelength (Å)	0.71073	0.71073
Crystal system, space group	Triclinic, *P*-1	Monoclinic, *P*2_1_/*c*
Unit cell dimensions	a = 8.879(2) Å α = 71.29(3)° b = 12.643(3) Å β = 80.79(3)° c = 15.062(3) Å γ = 78.58(3)°	a = 17.1322(10) Å α = 90° b = 12.7920(5) Å β = 94.461(4)° c = 14.6083(7) Å γ = 90°
Volume (Å^3^)	1561.2(7)	3191.8(3)
Z	2	4
Calculated density (Mg m^−3^)	2.337	1.697
Absorption coefficient (mm^−1^)	11.345	5.742
*F*(000)	1028	1616
Crystal size (mm)	0.15 × 0.14 × 0.13	0.17 × 0.13 × 0.11
Theta range for data collection (°)	2.55 to 29.278	3.219 to 29.509
Limiting indices	−12 ≤ h ≤ 12 −17 ≤ k ≤ 17 −20 ≤ l ≤ 20	−20 ≤ h ≤ 23 −16 ≤ k ≤ 17 −20 ≤ l ≤ 20
Reflections collected/unique	24,229/8402 [*R*_int_ = 0.0747]	19,630/8551 [*R*_int_ = 0.0520]
Completeness to theta (%)	99.9	98.8
Refinement method	Full-matrix least-squares on *F*^2^	
Data/restraints/parameters	8402/0/481	8551/2/377
Goodness-of-fit on *F*^2^	1.066	1.05
Final R indices [*I* > 2σ(*I*)]	*R*_1_ = 0.0718, *wR*_2_ = 0.1867	*R*_1_ = 0.0631, *wR*_2_ = 0.1629
R indices (all data)	*R*_1_ = 0.1077, *wR*_2_ = 0.2158	*R*_1_ = 0.0880, *wR*_2_ = 0.1827
Largest diff. peak and hole (e Å^−3^)	3.404 and −4.129	2.368 and −2.488

## Supplementary Materials

Supplementary materials can be found at https://www.mdpi.com/1422-0067/21/22/8696/s1. Table S1. Selected bands of the most important bonds in the FT-IR spectra of pyridine-2,3-dicarboxylic acid and Bi(III) polymers **1** and **2** (cm^−1^). Figure S1. The UV-Vis spectrum of the product of the reaction of BiCl_3_ with H_2_O in 1M HCl solution.
