# Supplementary material for: Structural Insights into New Bi(III) Coordination Polymers with Pyridine-2,3-Dicarboxylic Acid: Photoluminescence Properties and Anti-Helicobacter pylori Activity
Source: Int J Mol Sci. 2020 Nov 18;21(22):8696. doi: 10.3390/ijms21228696 (PMC7698728; doi:10.3390/ijms21228696)
Supplement: Supplementary file 1 [file ijms-21-08696-s001.pdf]

Structural insights into new Bi(III) coordination polymers with pyridine-2,3-dicarboxylic acid: Photoluminescence properties and anti-*Helicobacter pylori* activity

Mateusz Kowalik, Joanna Masternak, Iwona Łakomska, Katarzyna Kazimierczuk, Anna Zawilak-Pawlik, Piotr Szczepanowski, Oleksiy V. Khavryuchenko, Barbara Barszcz

Table S1. Selected bands of the most important bonds in the FT-IR spectra of pyridine-2,3-dicarboxylic acid and Bi(III) polymers **1** and **2** (cm^{-1}).

Assignments	$2,3pydcH_2$	1	2
$\nu(O–H)_{H2O}$	–	3600–3200 *br*	–
$\nu(N–H)_{pyH}$	3103	–	–
$\nu(N–H)_{Et3NH}$	–	–	2712, 2511 *br*
$\nu(C=O)_{COOH}$	–	1727	1708
$\nu_{as}(COO)$	1581	1618, 1574, 1537	1618, 1563
$\nu_s(COO)$	1361	1372	1381, 1369
$\Delta\nu$	220	246, 202, 165	249, 182
$\nu(C=C/N)$	1600, 1469, 1408	1605, 1449	1573, 1479, 1441
$\nu(C–N)_{Et3NH}$	–	–	1272

$\Delta\nu = \nu_{as}(COO) – \nu_s(COO)$; *br* – broad; pyH – pyridinium cation; Et_3NH –triethylammonium cation.

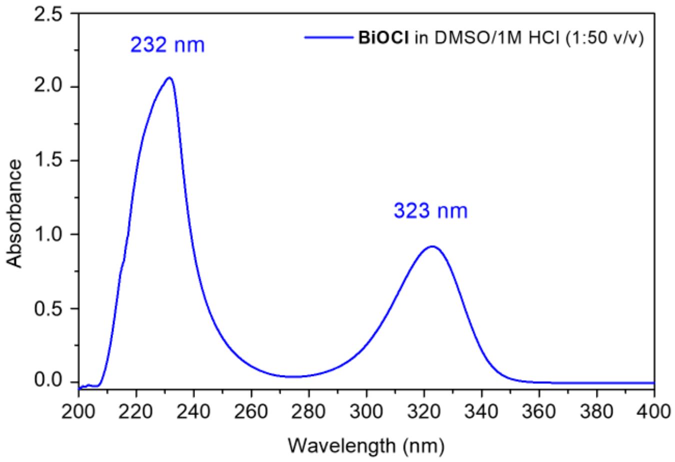

Fig. S1. The UV-Vis spectrum of the product of the reaction of $BiCl_3$ with H_2O in 1M HCl solution.
